# A transgenic female killing system for the genetic control of *Drosophila suzukii*

**DOI:** 10.1038/s41598-021-91938-1

**Published:** 2021-06-21

**Authors:** Marc F. Schetelig, Jonas Schwirz, Ying Yan

**Affiliations:** 1 Institute for Insect Biotechnology, Department of Insect Biotechnology in Plant Protection, Justus-Liebig-University Giessen, Winchesterstraße 2, 35394 Giessen, Germany; 2 Fraunhofer Institute for Molecular Biology and Applied Ecology IME, Winchesterstraße 2, 35394 Giessen, Germany

**Keywords:** Synthetic biology, Genetic engineering, Molecular engineering, Entomology

## Abstract

The spotted wing Drosophila (*Drosophila suzukii*) is an invasive pest of soft-skinned fruit crops. It is rapidly transmitted in Europe and North America, causing widespread agricultural losses. Genetic control strategies such as the sterile insect technique (SIT) have been proposed as environment-friendly and species-restricted approaches for this pest. However, females are inefficient agents in SIT programs. Here we report a conditional female-killing (FK) strategy based on the tetracycline-off system. We assembled sixteen genetic constructs for testing in vitro and in vivo. Twenty-four independent transgenic strains of *D. suzukii* were generated and tested for female-specific lethality. The strongest FK effect in the absence of tetracycline was achieved by the construct containing *D. suzukii nullo* promoter for early gene expression, *D. suzukii* pro-apoptotic gene *hid*^*Ala4*^ for lethality, and the *transformer* gene intron from the Mediterranean fruit fly *Ceratitis capitata* for female-specific splicing. One strain carrying this construct eliminated 100% of the female offspring during embryogenesis and produced only males. However, homozygous females from these FK strains were not viable on a tetracycline-supplemented diet, possibly due to the basal expression of *hid*^*Ala4*^. Potential improvements to the gene constructs and the use of such FK strains in an SIT program are discussed.

## Introduction

The spotted wing Drosophila (*Drosophila suzukii*; Diptera, Drosophilidae) is an invasive pest that has recently emerged as a global threat to fruit production and trade^1,2^. *D. suzukii* is challenging to control with pesticides because the larvae burrow inside ripening fruit and are not affected by chemicals on the surface^3,4^. Alternative, environmentally sustainable control measures such as the sterile insect technique (SIT) have been proposed or investigated for this pest^5,6^. SIT involves the release of mass-produced radiation-sterilized insects into the designated area and works on the basis that mating between sterile males and wild, fertile females produces no offspring^7,8^. SIT has been used as an area-wide integrated pest management (AW-IPM) strategy globally to combat agricultural pests and human disease vectors^9,10^. However, females are inefficient SIT agents because they compete with wild females for sterile males, thus reducing the effectiveness of the strategy^7,11^. The Female-killing (FK) strategies using the binary tetracycline-off (Tet-off) system were first introduced in *Drosophila melanogaster* to eliminate females adults^12,13^. The transgenic sexing strains (TSSs) based on female-specific splicing were developed in different insect species. Most of the TSSs eliminated females at the pupal stage, supposedly due to a transcriptional squelching^14,15^. Since the rearing of females contributes significantly to the running costs of SIT programs^16,17^, transgenic embryonic sexing systems (TESS) have been established in several fruit fly species^18–20^ and livestock pests^21–23^ to kill all females during embryogenesis and save rearing costs. TESS often takes advantage of transposable elements incorporating the Tet-off system, allowing the generation of transgenic insect strains to be reared on diets supplemented with tetracycline. This drug binds to the tetracycline transactivator (tTA) protein and prevents its interaction with the tetracycline response element (*TRE*) in the genetic construct^24^. When tetracycline is removed, a lethal gene linked to the *TRE* is activated only in females due to the presence of an intron with a sex-specific splicing pattern, thus eliminating all female embryos produced under laboratory and mass-rearing conditions^18,19,22,23^. In theory, such FK strategies are more effective than the SIT approach because fewer insects are required for a shorter period of time to achieve the same suppression effect^11,25^.

The tTA can be expressed under the control of cellularization gene promoters (driver cassette), which are most active during the early blastoderm stage, thus ensuring embryonic lethality^26^. The female-specific intron from a sex determination gene such as *transformer* (*tra*) is typically placed within a pro-apoptotic gene (effector cassette) to ensure that only females are killed^18,19,22,23^. We have previously reported the isolation and analysis of *D. suzukii* cellularization gene promoters, pro-apoptotic genes, and the nuclear localization signal (NLS) of the *tra* gene from the standard USA laboratory strain^27–29^. In these studies, we confirmed the embryonic expression of the cellularization genes *nullo, serendipity-α* (*sry-α*), *bottleneck* (*bnk*), and *slow-as-molasses* (*slam*) and the functionality of the pro-apoptotic genes *head involution defective* (*hid*), *grim,* and *reaper* (*rpr*)*.* Here, we generated 16 all-in-one (AIO) *piggyBac* plasmids containing the driver and effector cassettes in a single genetic construct. Some of these constructs were tested in vitro for their effect on cell survival, and the most promising ones were used to generate transgenic *D. suzukii* strains. We also measured *tTA* expression levels and determined the developmental stage of lethality for females in the absence of tetracycline in several FK strains.

## Results

### Construct design and cell culture experiments

**Figure 1:**
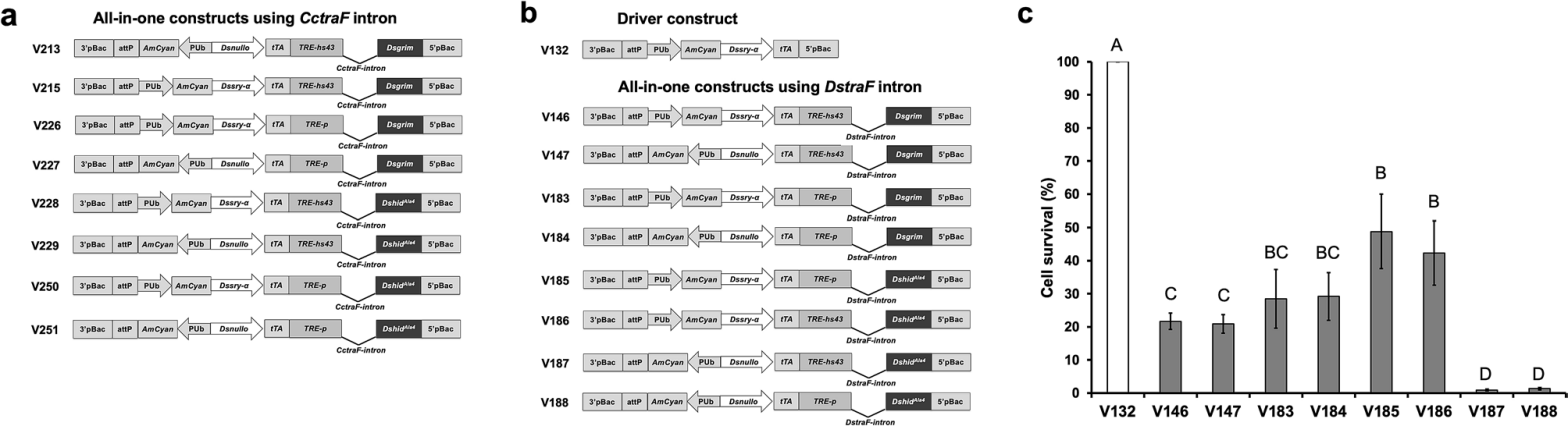
Female-killing genetic constructs and cell culture analysis. Schematic map of the all-in-one (AIO) *piggyBac* vectors, all of which comprise driver and effector cassettes based on the tetracycline-off system. The AIO constructs contain the female-specific *transformer* (*tra*) intron from (**a**) *C. capitata* (*CctraF*), or (**b**) *D. suzukii* (*DstraF*). All constructs harbor the AmCyan marker gene controlled by the constitutive *D. melanogaster polyubiquitin* (PUb) promoter and an *attP* recombination site. (**c**) AsE01 cells were co-transfected with pIE4-EGFP and one of the AIO constructs containing *DstraF*. The number of cells showing green fluorescence was counted using Image J. The cell survival (%) was calculated by dividing the number of EGFP positive cells from each construct by the number of EGFP positive cells from the control construct (V132). Each bar presents the mean ± SE of n = 3 experiments. Different letters in (c) indicate significant differences at *P* < 0.05 (one-way ANOVA, Holm-Šidák method).

We generated a range of AIO lethal constructs for in vitro cell culture assays and in vivo analysis, each containing the AmCyan marker gene controlled by the constitutive *D. melanogaster polyubiquitin* (PUb) promoter and an *attP* recombination site (Fig. 1a,b). Each AIO construct consisted of a driver cassette in which either the *Dssry-α* or *Dsnullo* promoter was placed upstream of the *tTA* coding region and an effector cassette containing either a phospho-mutated version of *hid* (*Dshid*^*Ala4*^) or the wildtype (WT) version of *grim* (*Dsgrim*). The effector cassettes also featured the *TRE* sequence fused to the minimal *P* or *hs43* promoter (*TRE-p* or *TRE-hs43*)^18^ and the female-specific splicing intron *CctraF* or *DstraF* (from *Ceratitis capitata* and *D. suzukii tra* genes, respectively) immediately downstream of the ATG translational start codon of the pro-apoptotic gene^18,21^. To evaluate the lethality of these constructs, the driver construct with the *Dssry-α* promoter (V132) and the AIO constructs with *DstraF* (Fig. 1b) were tested in AsE01 cells, originally derived from embryos (aged 20 h) of the Caribbean fruit fly *A. suspensa*^30^. AsE01 cells were co-transfected with pIE4-EGFP and one of the test constructs, and the cell survival (%) was calculated by dividing the number of EGFP positive cells from each test construct by the number of EGFP positive cells from the control construct (V132). The results suggested that all the AIO constructs significantly reduced cell survival compared to V132 (*P* < 0.001, One-way ANOVA). With V132 set to 100% survival, the AIO constructs fell into three broad groups defined by lethality. V185 and V186 represented the least effective group (49% and 42% cell viability, respectively), whereas V146, V147, V183, and V184 were moderately effective (22%, 21%, 28%, and 29% cell viability, respectively). The most lethal constructs were V188, with 1.3% viability, and V187, with 0.9% survival (Fig. 1c).

### Germline transformation and female lethality tests

In the first round of injections, five AIO constructs containing the *DstraF* intron were used for *piggyBac*-mediated germline transformation, resulting in two independent transgenic lines from V146, five from V183, six from V185, and one from V188 (Table S1). The transgene had inserted into the X chromosome in one of the V183 lines (F36m1), whereas all other lines were autosomal transformants. Most lines were bred to homozygosity on standard tetracycline diet (100 µg/ml) before female lethality tests, but V188 (M11m2) was weak and produced few homozygous offspring, and was therefore tested for female lethality using heterozygous flies. In the absence of tetracycline, the proportion of female offspring in these lines was 47.2–58.2% (*P* > 0.05, One-way ANOVA, Table 1), suggesting that the *DstraF* intron was inactive or the transgene was not expressed in adequate levels to induce lethality in females.

**Table 1:** Female lethality tests for different transgenic *D. suzukii* lines.

Driver^a^	Effector^b^	Strains	# Copy^c^	Tet^d^	# Eggs^e^	# Pupae	# Males	# Females	% Females^f^
−	−	WT-USA	−	−	971 ± 167 ab	767 ± 208 a	275 ± 118 ab	300 ± 104 a	53.5 ± 4.6 ab
				+	1356 ± 374 ab	750 ± 142 a	274 ± 90 ab	291 ± 84 a	52.3 ± 3.6 ab
*Dssry-α-* *tTA*	*TREhs43-Dsgrim-DstraF*	V146_M5f1	2	−	742 ± 29 bc	427 ± 23 ab	163 ± 14 ab	188 ± 18 ab	53.6 ± 2.3 ab
		V146_M9f1		−	755	433	174	160	47.9
*Dssry-α-* *tTA*	*TREp-Dsgrim-DstraF*	V183_M11m1	2	−	759	291	98	144	59.5
		V183_ F1m1		−	462	145	52	56	51.9
		V183_ F5m1		−	834	355	107	148	58.0
		V183_ F23f1		−	277	81	35	45	56.3
		V183_ F35m1		−	1353 ± 137 a	594 ± 154 ab	231 ± 39 a	243 ± 58 a	51.3 ± 2.5 ab
				+	1367 ± 62 a	657 ± 288 ab	254 ± 106 ab	254 ± 109 a	49.9 ± 1.0 ab
*Dssry-α-* *tTA*	*TREp-Dshid*^*Ala4*^*-DstraF*	V185_M1m1	2	−	376 ± 171 c	182 ± 38 c	62 ± 17 b	78 ± 23 bc	59.8 ± 2.2 a
				+	391 ± 62 c	179 ± 103 bc	52 ± 5 b	70 ± 36 bc	55.1 ± 10.7 ab
		V185_ F16f1		−	406	220	79	80	50.3
		V185_ F20m1		−	358	211	85	77	47.5
		V185_ F22f1		−	253	55	23	32	58.2
		V185_ F25m1		−	404	166	67	60	47.2
		V185_ F29f1		−	453	159	59	64	52.0
*Dsnullo-* *tTA*	*TREp-Dshid*^*Ala4*^*-DstraF*	V188_M11m2	1	−	322	54	11	14	56.0
*Dssry-α-* *tTA*	*TREhs43-Dsgrim-CctraF*	V215_M8f5	2	−	783 ± 210 bc	347 ± 56 ab	122 ± 19 ab	134 ± 22 ab	52.5 ± 0.5 ab
				+	777 ± 131 bc	378 ± 66 ab	166 ± 40 ab	161 ± 36 ab	49.3 ± 2.5 ab
*Dssry-α-* *tTA*	*TREp-Dsgrim-CctraF*	V226_ F4m2	2	−	954 ± 113 ab	327 ± 147 ab	133 ± 49 ab	134 ± 37 ab	50.9 ± 4.0 ab
				+	873 ± 249 ab	227 ± 16 bc	91 ± 7 ab	114 ± 8 ab	55.6 ± 0.3 ab
*Dsnullo-* *tTA*	*TREp-Dsgrim-CctraF*	V227_M5F1	2	−	728 ± 84 bc	310 ± 70 bc	115 ± 27 ab	93 ± 15 bc	45.1 ± 0.2 b
*Dsnullo-* *tTA*	*TREhs43-Dshid*^*Ala4*^*-CctraF*	V229_M4f1	1	−	706 ± 70 b	345 ± 63 ab	160 ± 34 ab	28 ± 12 c	14.2 ± 3.4 c

^a^The driver cassette contains the *Dssry-α* or *Dsnullo* promoter for the regulation of *tetracycline transactivator* (*tTA*).

^b^The effector cassette contains the minimal promoter *p* or *hs43* fused to TRE, the pro-apoptotic gene *Dsgrim* or *Dshid*^*Ala4*^, and the female-specific splicing intron *DstraF* or *CctraF.*

^c^The tested strains carry either one copy (heterozygous) or two copies (homozygous) of the transgene.

^d^The tests were carried out with (+) or without (−) tetracycline (Tet).

^e^Data are shown as mean ± standard deviation for three biological replicates. Data without standard deviations represent one biological replicate.

^f^ The data in the same column followed by different lower-case letter are significantly different (*P* < 0.05, one-way ANOVA, Holm-Šidák method).

In the second round of injections, we used the AIO constructs containing the *CctraF* intron. Constructs V215, V226, V227, and V229 each generated one autosomal transgenic line. Homozygous flies from lines V226_F4m2, V215_M8f5, and V227_M5F1 were viable and fertile on standard tetracycline diet and produced similar numbers of male and female offspring when switched to diets without tetracycline. Homozygous males from line V229_M4f1 were viable, but all homozygous females died before the adult stage on the standard tetracycline-supplemented diet. Lethality tests in heterozygous V229_M4f1 flies showed that only 14.9% of the newly emerged adults were females, which was significantly different to WT (52.2%) under restrictive tetracycline conditions (*P* < 0.001, One-way ANOVA, Table 1). Therefore, the constructs V229 and V251 were selected for the third round of injections, featuring the *Dsnullo* promoter, *Dshid*^*Ala4*^ effector gene, and *CctraF* intron differing only in the nature of the minimal promoter linked to *TRE* (Fig. 1a). We injected another 1795 embryos with V229, from which 64 fertile G_0_ flies (22 females and 42 males) were crossed, and six autosomal lines were recovered. Notably, all these V229 transgenic lines were from the G_0_ male crosses, suggesting that the transient expression of V229 killed the G_0_ females. We injected another 1495 embryos with V251, yielding only 21 fertile G_0_ adults. No transgenic flies were obtained from these G_0_, and the fertile eclosion rates were much lower than other constructs suggesting the transient expression of V251 may be toxic (Table S1).

A meta-analysis suggested that most *piggyBac* constructs for insect applications are 10,000–15,000 bp in length^31^. Here, the size of AIO constructs fell within the range 10,503–14,818 bp and the transformation frequencies were 3.4–11.6%, which is lower than the 16% that previously reported^32^. Increased donor and helper concentrations for V229 were associated with a reduced hatch rate of 27.1 to 17.5% and an increased transformation frequency of 3.8 to 9.4% (Table S1). All lines generated from the first and second round of injections produced fewer adult offspring than those of WT flies, possibly reflecting the fitness cost of the transgene (Table 1). Specifically, there was no significant difference in the number of male offspring between WT and any of the transgenic strains (*P* > 0.05, One-way ANOVA), whereas the WT strain produced significantly more female offspring than those from V185_M1m1, V227_M5F1, and V229_M4f1 under restrictive tetracycline conditions (*P* < 0.05, One-way ANOVA). In addition, the ratio of female offspring from V229_M4f1 was significantly lower than those from V185_M1m1 and V227_M5F1 (*P* < 0.001, One-way ANOVA). As observed for V229_M4f1, we were unable to breed the other V229 lines to homozygosity because homozygous females were non-viable on standard tetracycline diet. To determine whether the unwanted female lethality in the V229 lines was caused by a tetracycline dose insufficient to completely inhibit tTA binding to *TRE* (100 µg/ml), we tested flies from lines V229_M4f1, V229_M8f2, and V229_M36m1 with different concentrations of tetracycline (250, 500 and 1000 μg/mL) as well as doxycycline (100, 250 and 500 μg/mL), the latter being more effective than tetracycline for the suppression of tTA-induced lethality^26,33,34^. However, no homozygous adult females were recovered under any of these conditions, suggesting that homozygous females died due to the basal expression of *Dshid*^*Ala4*^. All V229 lines were therefore maintained by crossing heterozygous flies on standard tetracycline diet in each generation.

### Staged-lethality tests for V229 lines

To evaluate the FK efficiency of each V229 line, the heterozygous males were crossed with WT females in the absence of tetracycline (Table 2). Half the offspring were expected to be WT with a 1:1 sex ratio, and the other half were expected to be transgenic flies (heterozygous) with a strongly male-biased sex ratio. Indeed, all crosses produced similar numbers of WT males and females (female percentages ranged between 48.1 and 53.4%, *P* > 0.05, One-way ANOVA), and the eclosion rates of WT flies were not significantly different in the range of 70.4–75.0% (*P* > 0.05, One-way ANOVA, Table 2). As expected, a considerably lower female ratio of transgenic flies (fluorescent) was produced compared to that of WT flies in each cross (*P* < 0.05, One-way ANOVA). For example, crosses of the three lines M37f2, M39m1, and M41f1 generated transgenic offspring with only 1.3%, 10.2%, and 1.9% females, respectively. Females died within three days after emergence (Table 2). The eclosion rates of transgenic flies from these crosses were 39.9%, 40.6%, and 31.3%, respectively, which was significantly lower than those of WT flies (*P* < 0.05, One-way ANOVA), suggesting that most females died at the pupal stage. Notably, crosses to M44m1 individuals produced only transgenic males on emergence day and the eclosion rates of transgenic (69.8%) and WT (73.8%) flies were not significantly different (*P* > 0.05), suggesting that females died before the pupal stage (Table 2). In addition, the number of emerged WT and transgenic male offspring that survived until the end of day 1 or day 3 was significantly different from the M39m1 cross (*P* < 0.05, One-way ANOVA), indicating a deleterious effect of the transgene on the males in this line.

**Table 2:** Heterozygous tests for different V229 lines.

Line	Offspring^a^	# Pupae^b^	# Males (D1)^c^	# Females (D1)	% Females (D1)	% Eclosion^d^	# Males (D3)	# Females (D3)	% Females (D3)
M8f2	Fluorescent	285 ± 32 b	114 ± 15 b	55 ± 10 c	32.7 ± 2.6 b	59.2 ± 3.2 a	98 ± 14 bc	41 ± 4 c	29.6 ± 2.6 b
	Non-fluorescent	353 ± 46 b	122 ± 16 b	127 ± 15 b	51.0 ± 4.0 a	70.7 ± 3.5 a	105 ± 13 b	116 ± 10 b	52.6 ± 3.2 a
M36m1	Fluorescent	363 ± 29 b	150 ± 18 ab	31 ± 7 c	16.9 ± 2.0 c	49.7 ± 3.2 b	134 ± 15 ab	9 ± 3 d	6.0 ± 1.4 c
	Non-fluorescent	586 ± 44 a	221 ± 36 a	214 ± 21 a	49.4 ± 2.1 a	74.1 ± 4.6 a	198 ± 24 a	192 ± 15 a	49.3 ± 1.6 a
M37f2	Fluorescent	259 ± 14 b	100 ± 6 b	1.3 ± 0.6 d	1.3 ± 0.7 e	39.3 ± 1.0 b	83 ± 11 bc	0 e	0 d
	Non-fluorescent	345 ± 32 b	117 ± 19 b	134 ± 14 b	53.5 ± 2.2 a	73.1 ± 11.4 a	84 ± 8b c	96 ± 13 b	53.3 ± 2.3 a
M39m1	Fluorescent	364 ± 46 b	133 ± 39 b	15 ± 5 c	10.1 ± 0.9 d	40.1 ± 7.3 b	111 ± 39 bc	0 e	0 d
	Non-fluorescent	608 ± 73 a	226 ± 53 a	229 ± 33 a	50.6 ± 2.7 a	74.7 ± 8.4 a	196 ± 49 a	208 ± 28 a	51.8 ± 3.1 a
M41f1	Fluorescent	224 ± 19 b	69 ± 8 b	1.3 ± 0.6 d	1.9 ± 0.6 e	31.3 ± 1.4 b	61 ± 8 c	0 e	0 d
	Non-fluorescent	345 ± 28 b	132 ± 31 b	122 ± 26 b	48.2 ± 0.9 a	73.2 ± 11.3 a	120 ± 25 bc	115 ± 22 b	49.0 ± 0.6 a
M44m1	Fluorescent	213 ± 22 b	145 ± 19 ab	0 d	0 e	69.7 ± 3.1 a	120 ± 17 bc	0 e	0 d
	Non-fluorescent	385 ± 49 b	114 ± 20 b	140 ± 36 b	49.0 ± 3.2 a	73.4 ± 4.7 a	121 ± 13 bc	128 ± 39 b	50.8 ± 4.7 a

^a^Five heterozygous males from each V229 line were crossed with 10 WT virgin females in the absence of tetracycline, and the number of fluorescent (transgenic) and non-fluorescent (WT) offspring were counted.

^b^Data (mean ± standard deviation, n = 3) in the same column followed by different lower-case letter are significantly different (*P* < 0.05, one-way ANOVA, Holm-Šidák method).

^c^The adult offspring were counted either on emergence day (D1) or 3 days after emergence (D3).

^d^The number of freshly emerged adults (D1) divided by the number of pupae.

**Figure 2:**
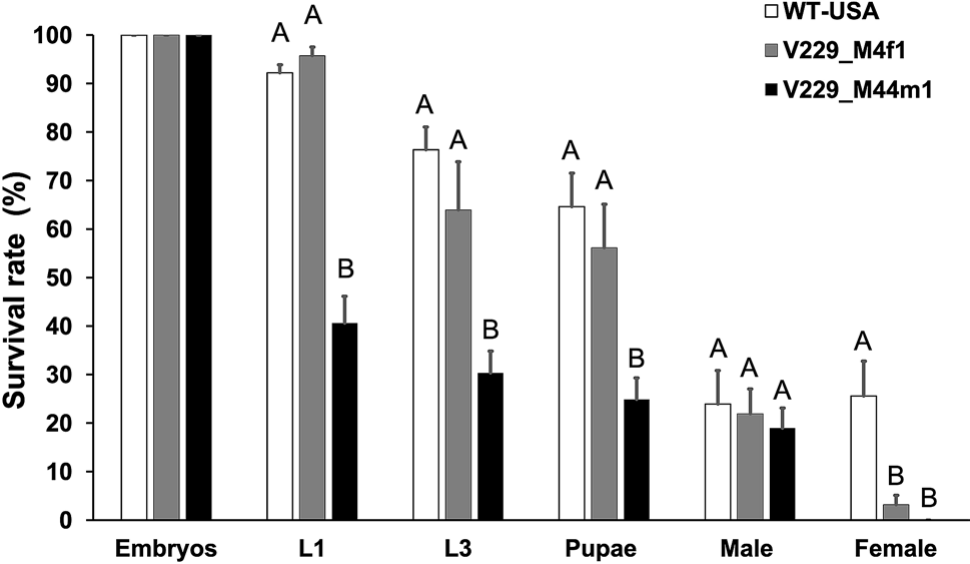
Determination of the lethal stage in transgenic *D. suzukii* lines. Homozygous males from lines V229_M4f1 or V229_M44m1 were crossed with wild-type (WT) virgin females on tetracycline-free diet, 500 embryos were collected, and we recorded the numbers of first-instar (L1) and third-instar (L3) larvae, pupae, adult males and adult females. Similar tests were conducted by crossing WT males and females as controls. The survival rate was calculated by dividing the number of flies in the corresponding stage or sex by the number of embryos. Each bar presents the mean ± SE of n = 7 experiments. Different letters indicate significant differences at *P* < 0.001 (one-way ANOVA, Holm-Šidák method).

To pinpoint the developmental stage of lethality in females, we carried out staged lethality tests in the absence of tetracycline by backcrossing the M4f1 and M44m1 lines individually to WT flies resulting in a strong FK effect (Fig. 2). From 3500 eggs each, 3228 WT and 3351 V229_M4f1 first-instar larvae were counted (no significant difference in the survival rate, *P* > 0.05, One-way ANOVA), indicating that V229_M4f1 females did not die during embryogenesis. Counted numbers of third-instar larvae, pupae and adult males from line V229_M4f1 were lower than those from WT, but not significantly different (*P* > 0.05, One-way ANOVA), but far fewer adult females (110) were generated from this line compared to WT (895) suggesting that V229_M4f1 females died at the pupal stage (*P* < 0.001, One-way ANOVA). In contrast, line V229_M44m1 produced 1421 first-instar larvae, approximately half the WT number (*P* < 0.001, One-way ANOVA), and only males developed from these larvae, indicating that V229_M44m1 females predominantly died at the embryonic stage (Fig. 2).

### The expression of *tTA* in different transgenic lines

**Figure 3:**
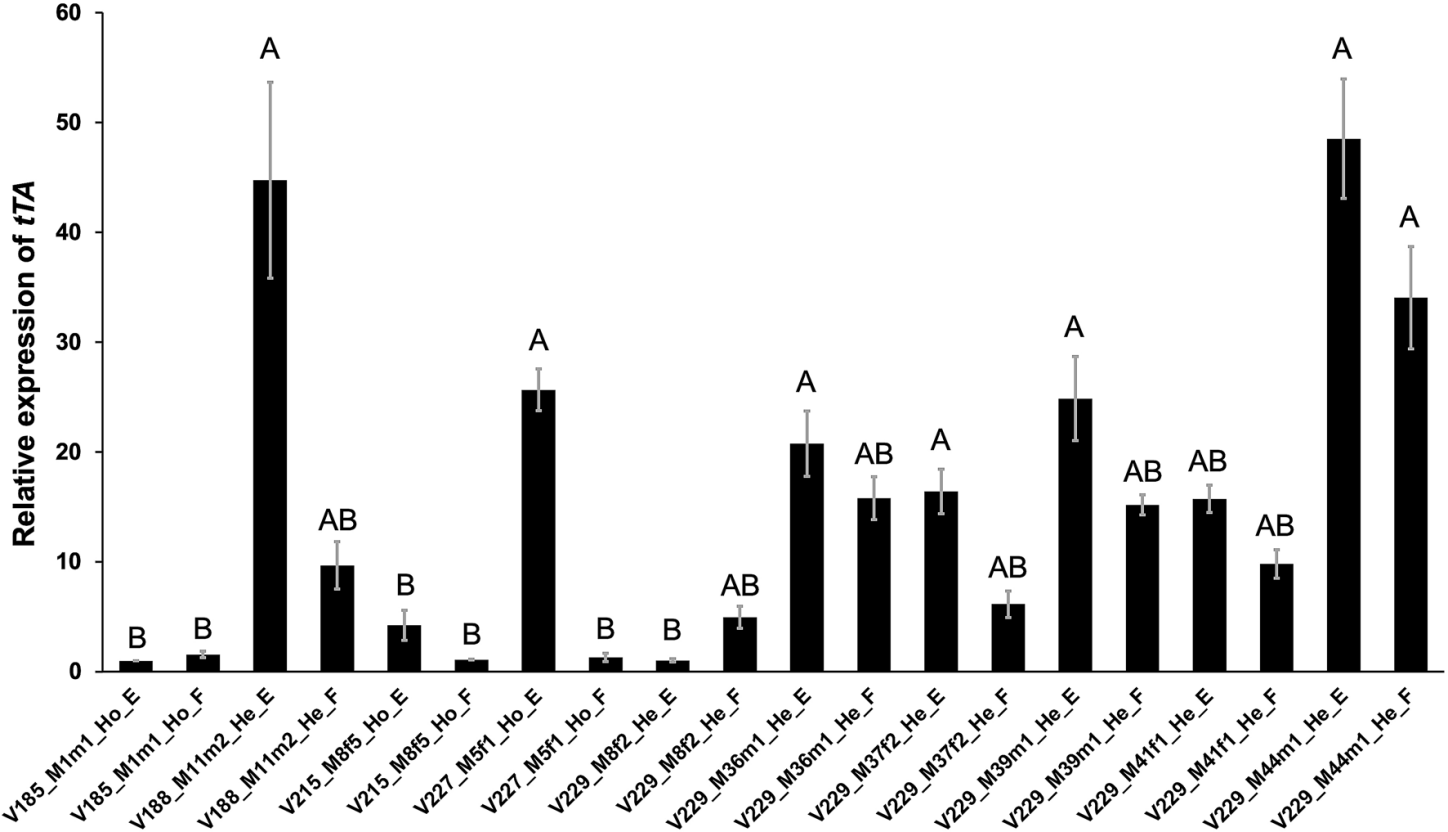
The *tTA* expression level in early embryos (E) and adult females (F) of different transgenic *D. suzukii* lines determined by quantitative real-time PCR. Homozygous (Ho) flies from lines V185, V215 and V227, and heterozygous (He) flies from lines V188 and V209 were compared. Gene expression was normalized to the reference gene *TBP* and is presented as a relative quantity based the *tTA* expression level in the embryos of line V185_M1m1. Data are presented as the mean ± SE from three replicate experiments. Different letters indicate significant differences at *P* < 0.05 (one-way ANOVA, Holm-Šidák method).

The *tTA* expression level in early embryos and female adults was determined by qRT-PCR, revealing higher *tTA* expression in the transgenic lines featuring the *Dsnullo* promoter compared to those featuring the *Dssry-α* promoter (Fig. 3). For example, *tTA* expression in V188_M11m2 (*Dsnullo-tTA*) heterozygous embryos was 44.7-fold higher (*P* < 0.001, One-way ANOVA) and in adult females was 6.1-fold higher (*P* = 0.036, One-way ANOVA) than in corresponding homozygous V185_M1m1(*Dssry-α -tTA*) individuals. Given that the V185 and V188 effector cassettes are identical (*TREp-DstraF_Dshid*^*Ala4*^), the difference in fitness between V185_M1m1 and V188_M11m2 (Table 1) must reflect either the toxicity of tTA accumulation^14^ or the induced expression of *Dshid*^*Ala4*^. Furthermore, *tTA* expression in V227_M5f1 (*Dsnullo-tTA*) embryos was 6.1-fold higher (*P* = 0.013, One-way ANOVA) than in V215_M8f5 (*Dssry-α -tTA*) embryos, but V227_M5f1 showed no FK effect (Table 1). This suggests that the V227 effector cassette (*TREp-CctraF_Dsgrim*) was inefficient at killing. Among the V229 lines, the highest *tTA* expression level was observed in M44m1 and the lowest in M8m2. Specifically, *tTA* expression in line V229_M44m1 embryos was 48.5-fold higher (*P* < 0.001, One-way ANOVA) and in females was 6.8-fold higher (*P* = 0.009, One-way ANOVA) than in corresponding individuals from line V229_M8m2. This was consistent with the lethality tests because V229_M44m1 was the only line that showed predominantly early female lethality whereas V229_M8m2 showed the weakest FK effect among the V209 lines (Table 2, Fig. 2).

## Discussion

The development of effective TESS constructs for *D. suzukii* benefits from the inclusion of homologous regulatory elements with predictable activity. Accordingly, we previously tested the promoters from four *D. suzukii* cellularization genes, revealing that the *Dsnullo* promoter achieved the strongest reporter gene expression in *D. melanogaster* S2 cells^28^. The *sry-α* promoter is also widely used for TESS constructs in fruit fly species^18–20^. We therefore selected the *Dsnullo* and *Dssry-α* promoters to control *tTA* expression in the driver cassette of our AIO constructs. Similarly, *Dsgrim* and *Dshid* were shown to be more potent pro-apoptotic genes than *Dsrpr* in S2 cell death assays^29^. We therefore used *Dsgrim* and *Dshid*^*Ala4*^, the latter predicted to be more lethal than the endogenous version^18,21^, in our effector cassette. Driver and effector plasmids using cellularization gene promoters or pro-apoptotic genes from *C. capitata* and *A. suspensa* were previously shown to kill AsE01 cells^35^. Here, we showed that our AIO plasmids also significantly reduce the survival of AsE01 cells, and that constructs containing the *Dsnullo* promoter and *Dshid*^*Ala4*^ (V187 and V188) were more cytotoxic than the other constructs (Fig. 1c). This is consistent with the in vivo tests, which showed that all transgenic lines generated using construct V229 (*Dsnullo* promoter + *Dshid*^*Ala4*^) killed females efficiently whereas lines featuring the *Dssry-α* promoter and/or *Dsgrim* were not female lethal (Tables 1 and 2). Indeed, higher *tTA* expression levels were observed in the transgenic *D. suzukii* lines featuring the *Dsnullo* promoter compared to the *Dssry-α* promoter (Fig. 3). These results indicate that the chosen *Dsnullo* promoter fragment expresses *tTA* to a higher level than the *Dssry-α* promoter, and *Dshid*^*Ala4*^ is a more effective lethal gene than *Dsgrim* in *D. suzukii*.

The *nullo* promoter has been used in the TESS construct for the Australian sheep blow fly *Lucilia cuprina*, in which all females died during embryogenesis^23^. In contrast, we found that the females of most V229 lines died at the pupal or adult stages, which means that feed would be wasted raising these females in a SIT program (Table 2, Fig. 2). The only exception was line V229_M44m1, in which all the females were eliminated during embryogenesis and *tTA* expression was highest among all the lines we tested (Fig. 2). The differences in *tTA* expression among different V229 lines probably reflected the chromosomal position effect, and it appears that a high level of *tTA* expression was needed to trigger the lethal dose of *Dshid*^*Ala4*^ at the early developmental stage. To achieve the rapid and strong production of tTA, other promoters from genes that are highly active at the pre-blastoderm stage could be considered for the driver cassette, such as genes involved in pattern formation^36^. The *TRE* from construct V229 comprises seven copies of the tTA binding site (tetracycline operator or *tet*O), so the performance of the effector cassette could be improved by using an alternative *TRE* with 21 *tet*O copies and a higher induction ratio^21,23^. These modifications could boost the production of *Dshid*^*Ala4*^ and thus kill females before the feeding stage. Another critical improvement needed for the effector cassette is to reduce the basal expression of *Dshid*^*Ala4*^ so females would be viable for homozygous breeding in the presence of tetracycline. A Drosophila synthetic core promoter (DSCP) has been developed in *D. melanogaster* with minimal leakage compared to the minimal *hsp70* promoter^37^. This could be used to replace the *hs43* or *P* minimal promoters in the effector cassette, ensuring the efficient suppression of *Dshid*^*Ala4*^ in the presence of tetracycline.

Female-specific splicing was induced by the *CctraF* intron in our V229 lines but the *DstraF* intron was unexpectedly inactive. Homologous regulatory elements usually outperform their heterologous counterparts when using the tetracycline-off system in transgenic organisms^38,39^. For example, *CctraF* and its counterpart from the New World screwworm fly *C. hominivorax* (*ChtraF*) were successfully used for female-specific splicing in both species because they share identical splice donor site and similar acceptor sites^19,40^. These sites are also highly conserved among several other dipteran species, suggesting they are important for sex-specific splicing^41^. We evaluated the *DstraF* and *CctraF* splice sites using a Drosophila splice site prediction program^42^ and multiple sequence alignments of endogenous genes and different gene constructs (Fig. S1). The results suggested that the exon sequences adjacent to the *traF* splice donor and acceptor sites may play a key role in splicing according to the genetic context, and a mismatch at this position between the endogenous *Dstra* (G-A) and our AIO constructs (G-G) may contribute to the inactivity of *DstraF* in our constructs (Fig. S1). For future development, *DstraF* could be placed between the adjoining G and A in the coding sequence of the effector gene. Furthermore, *traF* with adjacent G-G in the exons (as found in *D. albomicans* and *D. innubila*; Fig. S1b) could be inserted at the same position as *DstraF* in V229 as a possible approach for female-specific splicing of the pro-apoptotic gene.

The FK strains were maintained under heterozygous conditions by manual screening because the lethality of the construct to homozygous females could not be suppressed using tetracycline. Such approach would be unfeasible in a mass-rearing program, therefore homozygous viable and stable FK strains are still needed for the potential SIT application in *D. suzukii*. Our FK strains carry a single AIO gene construct at one locus, while the TESS strains in other insect species often have two constructs (drive and effector) located at two, separate locuses^18,19,21,22^. Population genetics model suggested that the two-construct strain can cause stronger population suppression compared to single construct strain^25^. Therefore, the driver and effector cassettes from the V229 vector can be arranged into two constructs which can be used to generate independent transgenic lines. By evaluating the activities of tTA and pro-apoptotic gene in these driver and effector lines, respectively, it should be possible to develop an FK strain without the leaky lethality by choosing driver and effector lines with moderate transgene activities^43^.

The release of fertile males carrying FK alleles was predicted to be more advantageous than sterile SIT males by mathematical modelling. This comes from the persistence of the FK effect through multiple generations from a transgene propagated via heterozygous males^11,25,44^. First greenhouse- and field-cage studies have been carried out for the FK strains of several insect species and the results supported the theoretical modeling and suggested that FK can be an effective method for population suppression^44–46^. However, the decision of releasing fertile strains should be carefully evaluated since the resistance to the lethality systems could arise. Recent studies showed that spontaneous mutations in a genetic strain could lead to the buildup of revertants, and in addition, a pre-existing inherent variation in the targeted field population could suppress the lethal system of an effector gene^47,48^. Nevertheless, FK approach can be used to produce male-only population and prevent the collateral damage to the fruits compared to a bi-sexual release, in which females can still damage fruits and lead to subsequent infestations even if they are sterilized^49,50^. Another technology in that direction was the development of a female to male sex reversal system in *D. suzukii* that was achieved by introducing a temperature-sensitive point mutation in the sex-determination gene *transformer-2* to reverse all females via heat shock to males^51^. However, male-only populations could not be obtained with this system because chromosomal XX females developed as sterile intersexuals, while XY males were sterile. Our study here characterized the performance of some key TESS components such as cellularization gene promoters, pro-apoptotic genes and sex-specific spliced introns, which could facilitate the development of future genetic control strategies for *D. suzukii* that require early or sex-specific gene expression or insect lethality and allow for the generation of male-only insect populations.

## Methods and materials

### Insect rearing and germ-line transformation

The wild-type (WT) *D. suzukii* USA strain and transgenic lines were maintained at 25 °C and 55–60% humidity with a 12-h photoperiod. The WT-USA strain was reared on tetracycline-free diet, and all transgenic strains were maintained on the same diet supplemented with 100 μg/mL tetracycline (Thermo Fisher Scientific). Flies were anesthetized with CO_2_ for screening and to set up crosses. Germ-line transformation with *piggyBac* constructs (Supplementary Material 1) was carried out as previously described^32^, except the eggs were collected from the WT-USA flies maintained on diets supplemented with 100 μg/mL tetracycline for at least 2 days before injection. A mixture of the *piggyBac* donor construct (500 or 700 ng/µl) and the *phsp-pBac* transposase helper (200 or 300 ng/µl) was injected into WT embryos. G_0_ adults were crossed to WT flies and offspring were screened for expression of the fluorescent marker at the pupal/adult stages. Segregation tests were conducted by outcrossing the transformants to WT flies, and independent homozygous strains were established by screening the fluorescence intensity at the third-instar larval stage for homozygous individuals. In some strains, homozygous females were not viable when the diet was supplemented with 100 μg/mL tetracycline and breeding was carried out with higher concentrations of tetracycline (250, 500, or 1000 μg/mL) or with doxycycline (Alfa Aesar) at concentrations of 100, 250 or 500 μg/mL.

### Cell culture experiments

The *Anastrepha suspensa* cell line UFENY-AsE01^30^ was cultivated in Schneider’s medium with 10% heat-inactivated fetal bovine serum (Hi-FBS) and 1% penicillin/streptomycin in closed-capped flasks without CO_2_ at 27.5 °C. Cells were passaged every 2 days until they reached ≥ 90% viability. For transient transfection, we used Xfectin reagent (Takara) according to the manufacturer’s instructions. Cells were seeded in 24-well plates at a density of 4 × 10^5^ live cells in 500 µl medium on 13-mm TC coverslips (SARSTEDT). After settling for 3 h, the cells were co-transfected with 0.5 µg *pIE4-EGFP*^52^ and 0.5 µg AIO or driver construct, 0.3 µl Xfectin, 28.7 µl Xfectin buffer and 270 µl serum-free Schneider’s medium. After incubation for 4 h at 27.5 °C, the medium was replaced with 500 µl Schneider’s containing 10% Hi-FBS and 1% penicillin/streptomycin and the cells were incubated for a further ~ 16 h at 27.5 °C. For visualization, cells were fixed in 4% paraformaldehyde (PFA) for 15 min and washed once with PBS. Cells expressing EGFP were imaged using an M205FA MZ FLIII microscope (Leica Microsystems) with EGFP filter sets (λ_excitation_ = 500/20; λ_emission_ = 535/30) and consistent settings. TC coverslips containing adhesive fluorescent cells were placed on a slide over a drop of Hi-FBS. We captured 10 images per coverslip, and counted the cells using Image J (Fiji) by first converting to 8-bit (threshold 35) inverted images, and then applying the watershed and automated cell count functions. The cell survival (%) was calculated by dividing the number of EGFP positive cells from each construct by the number of EGFP positive cells from the control construct (V132).

### Female lethality and staged lethality tests

To test the female lethality of homozygous AIO strains, newly emerged males and females were collected and reared separately on a tetracycline-free diet. Five males and 10 virgin females (3–5 days old) were then crossed in a large food vial (tetracycline-free, 175 ml volume, 50 × 100 mm), and the number of eggs was recorded every day during the transfer of flies into a new vial with fresh tetracycline-free diet for a further 7 days. We then counted the number of pupae and newly emerged adult males and females. For AIO strains with weak or nonviable homozygous females, we tested female lethality in a similar manner by crossing five homozygous males and 10 WT virgin females. For AIO strains in which female killing occurred at later developmental stages, female lethality was tested by crossing five heterozygous males and 10 WT virgin females, and then counting the number of fluorescent and non-fluorescent offspring at the pupal and adult (1 or 3 days after emergence) stages. One or three replicates were used for the female lethality test of each strain. The staged lethality tests were conducted by crossing WT or transgenic homozygous males with WT virgin females on tetracycline-free diet, and 500 eggs were collected on the glass slide, overlaid with halocarbon oil 700 (Sigma-Aldrich) and placed in the oxygen chamber. The hatched larvae were counted and transferred to the small food vial (50 ml volume, 29 × 95 mm; up to 50 larvae per vial) without tetracycline, and we scored the number of third-instar larvae, pupae, newly emerged adult males and females. For each transgenic or WT strain, the staged tests were carried out seven times.

### Quantitative real-time PCR (qRT-PCR)

Total RNA was isolated from embryos (2–6 h) or 5-day-old females using the Monarch Total RNA Miniprep Kit (New England Biolabs), and 0.5 μg was used for cDNA synthesis with the QuantiTect Reverse Transcription Kit (Qiagen). Homozygous flies from lines V185, V215 and V227 were used for RNA extraction, whereas heterozygous flies from lines V188 and V209 were used due to homozygous lethality. The QuantiNova SYBR Green RT-PCR Kit (Qiagen) was used for qPCR in a CFX96 Touch Real-Time PCR Detection System (Bio-Rad). Each reaction was heated to 98 °C for 3 min followed by 40 cycles of 98 °C for 10 s and 60 °C for 30 s. The primers are listed in Table S2. Three biological and three technical replicates were carried out for each reaction, and results were normalized to the *TATA binding protein* (*TBP*) reference gene using the 2^-∆∆Ct^ method as previously described^28,29^.

### Statistical analysis

Statistical analysis was carried out using SigmaPlot (v14.0). Differences in the post-transfection survival of AsE01 cells (cell count) expressing different constructs, in the survival rate, eclosion rate, fly number or female ratio of different insect strains for the staged or female lethality tests, and in the *tTA* expression levels of different transgenic lines, were analyzed by one-way analysis of variance (ANOVA) and means (log or square root transformed) were separated using the Holm-Šidák method.

## Supplementary Information

Supplementary Information 1.

Supplementary Information 2.

## Data Availability

All data generated or analyzed during this study are included in this published article [and its supplementary information files].
