# Supplementary material for: A transgenic female killing system for the genetic control of Drosophila suzukii
Source: Sci Rep. 2021 Jun 21;11:12938. doi: 10.1038/s41598-021-91938-1 (PMC8217240; doi:10.1038/s41598-021-91938-1)
Supplement: Supplementary file 1 — Supplementary Information 1. [file 41598_2021_91938_MOESM1_ESM.docx]

**A transgenic female-killing system for the genetic control of *Drosophila suzukii***

Marc F. Schetelig^1,2,3^, Jonas Schwirz^2,3^ and Ying Yan^1,2, *^

^1^ Justus-Liebig-University Giessen, Institute for Insect Biotechnology, Department of Insect Biotechnology in Plant Protection, Winchesterstraße 2, 35394 Giessen, Germany

^2^ Fraunhofer Institute for Molecular Biology and Applied Ecology IME, Winchesterstraße 2, 35394 Giessen, Germany

^3^ Co-first authors

^*^ Corresponding author

Correspondence and requests for materials should be addressed to Ying Yan.

Email: Ying.Yan@agrar.uni-giessen.de

Supplementary Material 1_Plasmid construction

For the driver cassettes, the *Dssry-α* promoter (211 bp) and *Dsnullo* promoter (2143 bp) were amplified from vectors *pCR4_Dssry-α* and *pCR4_Dsnullo* (Yan et al., 2020) using the primers listed in Table S2. The promoters were inserted into vector #1215_*pSLaf_tTA-SV40_af* (Schetelig et al. 2009) at the EcoRV and XbaI restriction sites (GeneArt seamless cloning) to yield constructs *V13_ pSLaf_Dssry-α-tTA-SV40* and *V14_ pSLaf_Dsnullo-tTA-SV40*, respectively. The promoter-tTA cassettes were isolated from V13 and V16 and transferred to *#1425_ pBXLII_attP220_PUbDsRed.T3-SV40* (Schetelig and Handler 2012) at the AscI/FseI sites to yield *V84_ pBXLII_attP_PUbDsRed.T3_Dssry-α-tTA-SV40* and *V86_ pBXLII_attP_PUbDsRed.T3_Dsnullo-tTA-SV40*, respectively. For the effector cassettes, the *att*P fragment was amplified from #1425 and inserted into *#1419_ pBXLII_PUb-EGFP-SV40* (Schetelig and Handler 2012) at the Bsp119I site to yield *V92_ pBXLII_attP_PUb-EGFP-SV40*. *Dsgrim* was amplified from *pCR4_Dsgrim* (Jaffri et al., 2020), the *D. suzukii* *transformer* female-specific intron (*DstraF*) was amplified from *pCR4_Ds-transformer-genomic* (Schwirz et al. 2020), the TRE-hsp43 amplified from *AH443*_ *pBXLII_PUbEGFP_TREhs43-CctraF-Alhid^Ala2^_loxN-3xP3-FRT-AmCyan_lox2272_loxP_attP235* (Schetelig and Handler 2012) and *SV40* amplified from #1425 were transferred to V92 by stepwise Gibson Assembly to yield *V127_ pBXLII_attP_PUb-EGFP_TREhs43-DstraF-Dsgrim-SV40*. An analogous strategy was used to generate *V129_ pBXLII_attP_PUb-EGFP_TREhs43-DstraF-DsHid^Ala4^-SV40*, containing *DsHid^Ala4^* amplified from *pCR4_DsHid^Ala4^* (Jaffri et al., 2020).

For the AIO constructs containing *DstraF*, the *PUb-DsRed-SV40* cassette from V84 was removed by Bsp119I digestion and replaced with the *PUb-AmCyan-SV40* cassette from *AH452_pBXLII_FRT_3xP3DsRed_FRT3_loxN-PUbAmCyan-lox2272* (Häcker et al. 2017) to obtain *V132_ pBXLII_attP_PUbAmCyan_Dssry-α-tTA-SV40* with the PUb and *Dssry-α* sequences in tandem. Similarly, the *PUb-AmCyan-SV40* cassette from AH452 was inserted into V86 at the Bsp119I site to obtain *V135_ pBXLII_attP_PUbAmCyan_Dsnullo-tTA-SV40* in which the PUb and *Dssry-α* sequences are not in tandem. The *TREhs43-Dsgrim-DstraInt-SV40* cassette was excised from V127 using AscI and blunted with Klenow, then inserted into V132 and V135 (linearized with EcoRV) to yield constructs *V146_ pBXLII_attP_PUbAmCyan_Dssry-α-tTA-SV40_TREhs43-Dsgrim-DstraF-SV40* and *V147_ pBXLII_attP_PUbAmCyan_Dsnullo-tTA-SV40_TREhs43-Dsgrim-DstraF-SV40*, respectively. The *p* promoter was amplified using primers P374 and P375, and was inserted into V146 and V147 at the BseRI and MluI sites to obtain *V183_ pBXLII_attP_PUbAmCyan_Dssry-α-tTA-SV40_TREp-Dsgrim-DstraF-SV40* and *V184_ pBXLII_attP_PUbAmCyan_Dsnullo-tTA-SV40_TREp-Dsgrim-DstraF-SV40*, respectively. The *DstraF-DsHid^Ala4^* cassette was excised from V129 and transferred to V146, V147, V183 and V184 at the MluI and BsmBI sites to obtain *V186_ pBXLII_attP_PUbAmCyan_Dssry-α-tTA-SV40_TREhs43-DsHid^Ala4^-DstraF-SV40*, *V187_ pBXLII_attP_PUbAmCyan_Dsnullo-tTA-SV40_TREhs43-DsHid^Ala4^-DstraF-SV40*, *V185_ pBXLII_attP_PUbAmCyan_Dssry-α-tTA-SV40_TREp-DsHid^Ala4^-DstraF-SV40* and *V188_ pBXLII_attP_PUbAmCyan_Dsnullo-tTA-SV40_TREp-DsHid^Ala4^-DstraF-SV40*, respectively.

For the AIO constructs containing *CctraF*, V146 and V147 were digested with MluI and BsmBI, and the vector backbone (without the *DstraInt-Dsgrim* cassettes) was purified. *CctraF* was amplified from AH443 using primers P492 and P493, and *Dsgrim* was amplified from *pCR4_Dsgrim* using primers P494 and P495, and the two fragments were inserted into the backbone of V146 and V147 by Gibson cloning to obtain *V213_ pBXLII_attP_PUbAmCyan_Dssry-α-tTA-SV40_TREhs43-Dsgrim-CctraF-SV40* and *V215_ pBXLII_attP_PUbAmCyan_Dsnullo-tTA-SV40_TREhs43-Dsgrim-CctraF-SV40*, respectively. Then the *Dsgrim-CctraF* cassette was excised from V215 and transferred to V183 and V184 at the MluI and BsmBI sites to generate *V226_ pBXLII_attP_PUbAmCyan_Dssry-α-tTA-SV40_TREp-Dsgrim-CctraF-SV40* and *V227_ pBXLII_attP_PUbAmCyan_Dsnullo-tTA-SV40_TREp-Dsgrim-CctraF-SV40*, respectively. Similarly, *CctraF* was amplified from AH443 using primers P492 and P602, and *DsHid^Ala4^* was amplified from *pCR4_DsHid^Ala4^* using primers P603 and P604. The two fragments were inserted into the backbone of V186 and V187 (digested with MluI and BsmBI) by Gibson cloning, to produce *V228_ pBXLII_attP_PUbAmCyan_Dssry-α-tTA-SV40_TREhs43-DsHid^Ala4^-CctraF-SV40* and *V229_ pBXLII_attP_PUbAmCyan_Dsnullo-tTA-SV40_TREhs43-DsHid^Ala4^-CctraF-SV40*, respectively. The *DsHid^Ala4^-CctraF* cassette was excised from V229 and transferred to V185 and V188 at the MluI and BsmBI sites to generate *V250_ pBXLII_attP_PUbAmCyan_Dssry-α-tTA-SV40_TREp-DsHid^Ala4^-CctraF-SV40* and *V251_ pBXLII_attP_PUbAmCyan_Dsnullo-tTA-SV40_TREp-DsHid^Ala4^-CctraF-SV40*, respectively.

**References**

Häcker I, Harrell Ii RA, Eichner G, Pilitt KL, O'Brochta DA, Handler AM, Schetelig MF (2017) Cre/lox-Recombinase-Mediated Cassette Exchange for Reversible Site-Specific Genomic Targeting of the Disease Vector, *Aedes aegypti*. Sci Rep. 7:43883. <https://doi.org/10.1038/srep43883>

Schetelig MF, Caceres C, Zacharopoulou A, Franz G, Wimmer EA (2009) Conditional embryonic lethality to improve the sterile insect technique in *Ceratitis* *capitata* (Diptera: *Tephritidae*). BMC Biol. 7:4. <https://doi.org/10.1186/1741-7007-7-4>

Schetelig MF, Handler AM (2012) A transgenic embryonic sexing system for *Anastrepha suspensa* (Diptera: *Tephritidae*). Insect Biochem Mol Biol. 42:790–795 <https://doi.org/10.1016/j.ibmb.2012.07.007>

Schwirz J, Yan Y, Franta Z, Schetelig MF (2020) Bicistronic expression and differential localization of proteins in insect cells and *Drosophila suzukii* using picornaviral 2A peptides. Insect Biochem Mol Biol. 119. <https://doi.org/10.1016/j.ibmb.2020.103324>
