# Supplementary material for: A transgenic female killing system for the genetic control of Drosophila suzukii
Source: Sci Rep. 2021 Jun 21;11:12938. doi: 10.1038/s41598-021-91938-1 (PMC8217240; doi:10.1038/s41598-021-91938-1)
Supplement: Supplementary file 2 — Supplementary Information 2. [file 41598_2021_91938_MOESM2_ESM.docx]

**A transgenic female-killing system for the genetic control of *Drosophila suzukii***

Marc F. Schetelig^1,2,3^, Jonas Schwirz^2,3^ and Ying Yan^1,2, *^

^1^ Justus-Liebig-University Giessen, Institute for Insect Biotechnology, Department of Insect Biotechnology in Plant Protection, Winchesterstraße 2, 35394 Giessen, Germany

^2^ Fraunhofer Institute for Molecular Biology and Applied Ecology IME, Winchesterstraße 2, 35394 Giessen, Germany

^3^ Co-first authors

^*^ Corresponding author

Correspondence and requests for materials should be addressed to Ying Yan.

Email: Ying.Yan@agrar.uni-giessen.de

Supplementary Material 2_ Supplementary tables and figure

**Table S1. *piggyBac*-mediated germ-line transformation in *D. suzukii***

| **Vector^a^ (size)** | **Injected eggs^c^** | **Hatched larvae** | **Fertile adults** | **Transgenic lines^d^** | **Transformation frequency^e^** | **Hatch**  **rate** | **Fertile eclosion rate (to larvae)^f^** |
| --- | --- | --- | --- | --- | --- | --- | --- |
| V146 (10,503 bp) | 481 | 173 | 20 | 2 | 10.0% | 36.0% | 11.6% |
| V183 (10,968 bp) | 753 | 305 | 43 | 5 | 11.6% | 40.5% | 14.1% |
| V184 (12,823 bp) | 640 | 167 | 17 | 0 | - | 26.1% | 10.0% |
| V185 (11,878 bp) | 802 | 346 | 53 | 6 | 11.3% | 43.1% | 15.3% |
| V188 (13,732 bp) | 631 | 285 | 55 | 1 | 1.8% | 45.2% | 19.3% |
| V213 (13,443 bp) | 538 | 107 | 27 | 0 | - | 19.9% | 25.2% |
| V215 (11,589 bp) | 626 | 173 | 29 | 1 | 3.4% | 27.6% | 16.8% |
| V226 (12,054 bp) | 410 | 51 | 9 | 1 | 11.1% | 12.4% | 17.6% |
| V227 (13,909 bp) | 493 | 122 | 12 | 1 | 8.3% | 24.7% | 9.8% |
| V228 (12,498 bp) | 310 | 97 | 13 | 0 | - | 31.3% | 13.4% |
| V229 (14,352 bp) | 339 | 92 | 26 | 1 | 3.8% | 27.1% | 28.2% |
| V250 (12,963 bp) | 378 | 129 | 28 | 0 | - | 34.1% | 21.7% |
| V251 (14,818 bp) | 413 | 70 | 10 | 0 | - | 16.7% | 9.7% |
| V229 (14,352 bp)^b^ | 1795 | 315 | 64 | 6 | 9.4% | 17.5% | 20.3% |
| V251 (14,818 bp)^b^ | 1495 | 248 | 21 | 0 | - | 16.6% | 8.5% |

^a^ Each vector (500 ng/µl) was co-injected with the *phsp-pBac* transposase helper plasmid AH286 (200 ng/µl).

^b^ Increased concentrations of vector (700 ng/µl) and helper AH286 (300 ng/µl) were used.

^c^ The eggs were collected from WT flies that were fed with tetracycline diet (100 µg/ml) for at least 2 days, and those injected eggs were also kept on the tetracycline diet afterwards.

^d^ Transgenic lines derived from independent G_0_ flies

^e^ Number of independent lines divided by number of fertile adults.

^f^ Number of fertile adults were divided by number of hatched larvae.

**Table S2. Primer sequences**

| **Primer No.** | **Primer Name** | **Primer Sequence** |
| --- | --- | --- |
| P39 | Dssry-α_F | CTGCGCGCGATCGATAGTTCTTTGCCTGTATTTCAG |
| P40 | Dssry-α_R | TTACTTTTATCTAATCTAGACATAGCTCTATAAGATGTGCTCC |
| P43 | Dsnullo_F | CTGCGCGCGATCGATGATGCTCTTTTAGGAATTTATC |
| P44 | Dsnullo_R | TTACTTTTATCTAATCTAGACATTTTTCTCAAAACTTAGGTT |
| P212 | attP-Bsp119I_F | ACTTTCGAAACTAGTACTGACGGAC |
| P213 | attP-Bsp119I_R | TCATTCGAACTGTACTAGTCGCGCTC |
| P179 | Dstra-intron-F | GCAGGCGGCCGCATGGTATATATACACATATTCGATCGG |
| P180 | Dstra-intron-R | CTACGTGGAAGTGGAAGAAGAAG |
| P214 | TRE-hs43-F | TAGATCGGCCGGCCTTGGCGCGCCTTGCTAGGCTGGCCGAATT |
| P178 | TRE-hs43-R | CATGCGGCCGCCTGCAGCT |
| P181 | Dshid^Ala4^-DstraInt-F | CTTCCACTTCCACGTAGGCCGTGCCCTTTTATTTGCCCG |
| P182 | Dshid^Ala4^-DstraInt-R | CACGTCTCCCGCGGTTTCATCGCGCCGCAAAGAAGC |
| P194 | Dsgrim-DstraInt-F | TTCTTCCACTTCCACGTAGGCCATTGCCTACTTCATACCC |
| P195 | Dsgrim-DstraInt-R | CACGTCTCCCGCGGTTTTAACTGCCGCTGCTGATTTCG |
| P220 | SV40-F | AACCGCGGGAGACGTGTAATAGCGGCCGCGACTCTAGATC |
| P215 | SV40-R | GTGAGTCGTATTAAGATCTAGGGATACATTGATGAGTTTGGACA |
| P374 | p-Promoter-F | ACATGCGGCCGCCTGCGATCGCTACGCGTGTGCTGGCTTCCTTTTTGTCCTTATCCGC |
| P375 | p-Promoter-R | ACATGAGGAGCGCCGGAGTAGCACGTTTGCTTGTTGAGAGG |
| P492 | CctraInt-F | CTAAACAATCCATCGCGAATCGACGTACGCGTATGGTAATTTTAAAAGCATATTTTTTTCTTTGAAATTC |
| P493 | CctraInt-Dsgrim-R | GGTCGGGTATGAAGTAGGCAATGGCCTATAGATACCATAGATGTATGGATTAGTATC |
| P494 | Dsgrim-CctraInt-F | GCCATTGCCTACTTCATACCCGACC |
| P495 | Dsgrim-CctraInt-R | CGCGGCCGCTATTACACGTCTCCCGCGGTTTTAACTGCCGCTGCTGATTTCG |
| P602 | CctraInt-Dshid^Ala4^-R | GCCGCCCTCGGGCAAATAAAAGGGCACGGCCTATAGATACCATAGATGTATGGATTAGTATC |
| P603 | Dshid^Ala4^-CctraInt-F | GCCGTGCCCTTTTATTTGCCCGAGG |
| P604 | Dshid^Ala4^-CctraInt-R | CGCGGCCGCTATTACACGTCTCCCGCGGTTTCATCGCGCCGCAAAGAAGC |
| P426 | tTA-qF | CAGAGCCAGCCTTCTTATTCG |
| P429 | tTA-qR | GGCCCTCGATGGTAGACC |
| P740 | DsTBP-qF | CCACGGTGAATCTGTGCT |
| P741 | DsTBP-qR | GGAGTCGTCCTCGCTCTT |


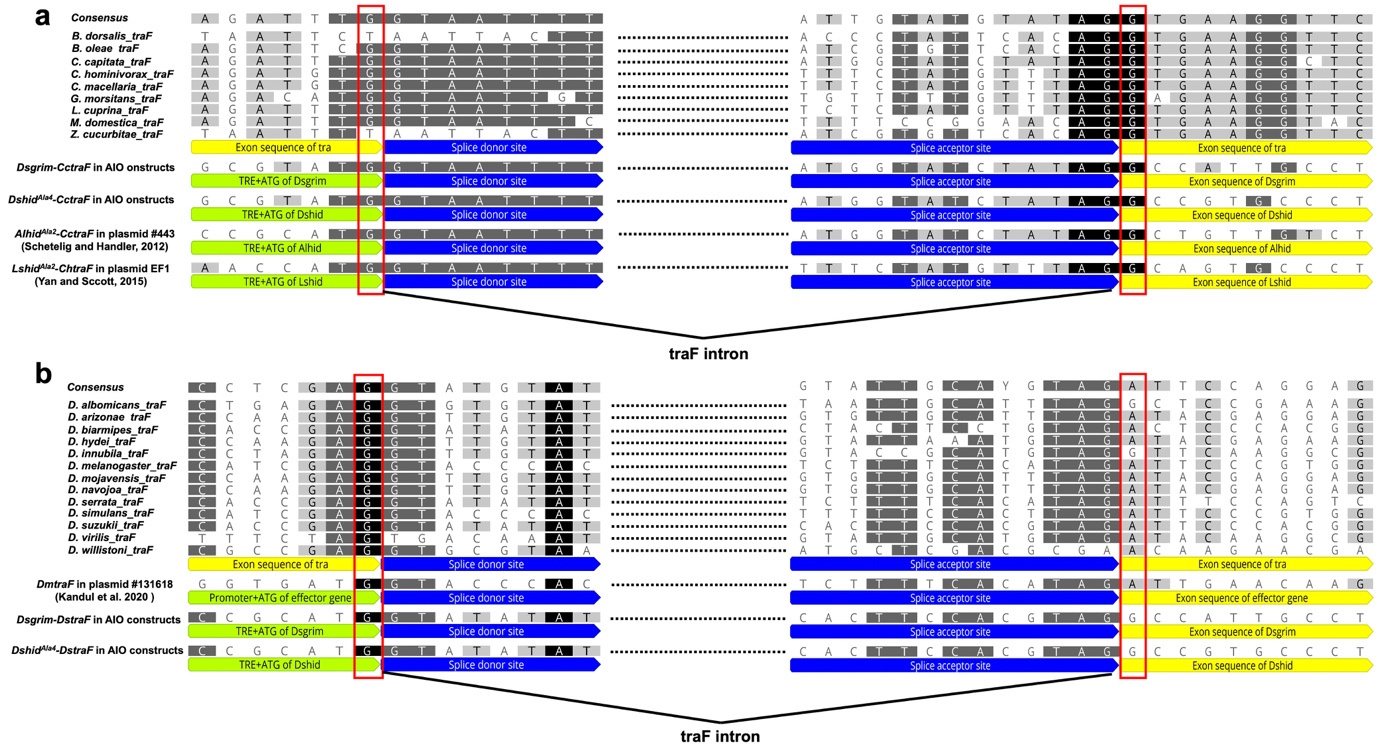


**Figure S1. Comparison of splicing sites in the female-specific intron of the *transformer* gene (*traF*) in different fly species and gene constructs.** The sequence information was downloaded from NCBI (<https://www.ncbi.nlm.nih.gov>) and the splice donor and acceptor sites were determined using the Drosophila splice site prediction program (NNsplice) (Li et al. 2013; Reese et al. 1997). (a). Sequence alignment to compare the splicing sites of *traF* from several dipteran species and plasmids. In the dipteran species, the exon sequence adjacent to the splice donor site is predominately “G” and the exon sequence adjacent to the acceptor site is always “G” (indicated by red box), suggesting that “G-G” at such positions may be important for the splicing of *traF* in these species. Indeed, *traF* from *C. capitata* (*CctraF*) and *C. hominivorax* (*ChtraF*) in plasmids 443# and EF1 follow the “G-G” model and successfully mediated female-specific expression of the effector gene *hid* in *A. suspensa* (Schetelig and Handler 2012) and *L. cuprina* (Yan and Scott 2015). Similarly, the exon sequences adjacent to the splice donor and acceptor sites of the *CctraF* in our AIO constructs are also “G-G”. In addition, *CctraF* received strong NNsplice prediction scores of 0.78 and 0.88 for splice donor and acceptor sites, respectively (0.4 is the cutoff for significance). Therefore, the high NNsplice score and the same arrangement of adjacent exon sequence “G-G” may partially explain why *CctraF_Dshid^Ala4^* efficiently mediated female-specific lethality in our V229 lines. (b). Sequence alignment to compare *traF* splicing sites in *Drosophila* species and a plasmid (Addgene, #131618) from a previous report (Kandul et al. 2020) and the AIO constructs in this study. For the Drosophila *traF* sequence, the exon sequence adjacent to the splice donor site is always “G” and the exon sequence adjacent to the acceptor site is predominately “A” (indicated by red box), suggesting that “G-A” at these positions is important for *traF* splicing in these species. Recently, *DmtraF* with “G-A” in the adjacent exon was successfully used for female-specific transgene expression in *D. melanogaster* (Kandul et al. 2020). Specifically, *DmtraF* was inserted into an effector gene after the start codon ATG in the plasmid #131618, forming a “G-A” pattern identical to that from the endogenous gene. *DmtraF* also received high NNsplice scores of 0.92 and 0.96 for splice donor and acceptor sites, respectively. On the other hand, *DstraF* received a score of 0.92 for the donor site but a much lower score of 0.57 for acceptor site, indicating that the acceptor site of *DstraF* is less preferred by the spliceosome. In addition, placing *DstraF* after the start codon of a pro-apoptotic gene in the AIO constructs resulted in a “G-G” pattern, differing from the “G-A” pattern of the endogenous gene. Consequently, the mismatch from “A” to “G” in the exon adjacent to the *DstraF* acceptor site in our AIO constructs may further weaken the preference of the spliceosome, which may explain the inactive *DstraF* in our effector cassettes.

**References**

Kandul NP, Liu J, Hsu AD, Hay BA, Akbari OS (2020) A drug-inducible sex-separation technique for insects. Nat Commun. 11:2106. <https://doi.org/10.1038/s41467-020-16020-2>

Li F, Vensko SPI, Belikoff EJ, Scott MJ (2013) Conservation and sex-specific splicing of the transformer gene in the Calliphorids *Cochliomyia hominivorax*, *Cochliomyia macellaria* and *Lucilia sericata*. PLoS ONE 8:e56303. <https://doi.org/10.1371/journal.pone.0056303>

Reese MG, Eeckman FH, Kulp D, Haussler D (1997) Improved splice site detection in Genie. J Comput Biol. 4:311-323. <https://doi.org/10.1089/cmb.1997.4.311>

Schetelig MF, Handler AM (2012) A transgenic embryonic sexing system for *Anastrepha suspensa* (Diptera: *Tephritidae*). Insect Biochem Mol Biol. 42:790–795 <https://doi.org/10.1016/j.ibmb.2012.07.007>

Yan Y, Scott MJ (2015) A transgenic embryonic sexing system for the Australian sheep blow fly *Lucilia cuprina*. Sci Rep. 5:16090. <https://doi.org/10.1038/srep16090>
